# Improving Informed Consent for Novel Vaccine Research in a Pediatric Hospital Setting Using a Blended Research-Design Approach

**DOI:** 10.3389/fped.2020.520803

**Published:** 2021-01-12

**Authors:** Sally M. Jackson, Margherita Daverio, Silvia Lorenzo Perez, Francesco Gesualdo, Alberto E. Tozzi

**Affiliations:** ^1^Multifactorial and Complex Diseases Research Area, Bambino Gesù Children's Hospital IRCCS, Rome, Italy; ^2^Libera Università Maria SS. Assunta, Rome, Italy; ^3^AND Consulting Group, Brussels, Belgium

**Keywords:** informed consent, ethics, mixed-methods, clinical research, natural language processing (nlp), design thinking (DT), vaccination, inclusion

## Abstract

It is necessary to conduct Clinical Trials in children, including for novel vaccines. Children cannot legally provide valid consent, but can assent to research participation. Informed consent and assent communications are frequently criticized for their lack of comprehensibility and often, researchers do not involve patients in informed consent design. We tested a blended research-design approach to co-design multimedia informed consent prototypes for experimental vaccine studies targeted at the pediatric population. We report details on the methodology utilized, and the insights, ideas, and prototype solutions we generated using social media data analysis, a survey, and workshops. A survey of clinical trial researchers indicated that while the most did not use technology for informed consent, they considered its utilization favorable. Social media analysis enabled researchers to quickly understand where community perspectives were concordant and discordant and build their understanding of the types of topics that they may want to focus on during the design workshops. Participatory design workshops for children and their families reaped insights, ideas, and prototypes for a range of tools including apps and websites. Participants felt that the prototypes were better able to communicate necessary content than the original text document format. We propose using a participatory, mixed-methods approach to design informed consent so that it is better adapted to patients' needs. Such an approach would be helpful in better addressing the needs of different segments of the populations involved in clinical trials. Further evidence should be gained about the impact of this strategy in improving recruitment, decreasing withdrawals and litigations, and improving patient satisfaction during clinical trials.

## Introduction

Legal attempts to improve transparency (1, 2) have increased the amount of information disclosed to research participants through informed consent documents, which have become increasingly lengthy and complex (3, 4). According to EU Regulation, informed consent needs to be concise to be understandable (2) yet comprehensiveness and conciseness are conflicting needs that can lead to poor communication at a time when potential research participants are making an important decision about their health (5, 6).

Clinical trials are frequently necessary in children at different growth and developmental stages due to children's diverse needs which differ to adults (7–13). As the ability to understand complex information evolves with age and children are less likely than adults to be able to express their needs and defend their interests, they are considered less able to give consent (12), and do not have the legal capacity to do it (2). Researchers are obliged to obtain consent from parents or legal representatives, and children can provide assent appropriate to their age and maturity (14). Researchers, legislators, parents, and children differ in opinion about which information is most relevant to include, as well as mechanism of delivery (15–17). Emotion and trust play a strong role in decision making, particularly with children (18).

Advances in technology have led to new opportunities to support the informed consent and assent process (19). Participatory methods have previously been proposed to involve patients in informed consent and assent design (20). To co-create informed consent with participants, we looked to “design thinking” (DT). DT is a user-centered (21, 22) participatory approach used to creatively address complex problems. It is increasingly being utilized in a wide range of health domains at the individual (e.g., disease prevention and health management) and the system levels (e.g., health care management and organizational change) (23–26).

DT is based upon three main pillars—empathy, collaboration, and experimentation (21). The priority that DT places on end-users' desires, needs, and challenges, results in a better understanding of the problem in order to develop more comprehensive and effective solutions (27). Multidisciplinary teams are compiled and then prompted to generate a large number of ideas which are honed, and prototypes are rapidly developed and tested (22, 28, 29). Rapid cycles of insight and idea generation, testing, and prototyping reduce the timeframe for design and implementation (30).

In mid-2019, we tested a blended research-design approach to co-design multimedia informed consent prototypes for experimental vaccine studies targeted at the pediatric population. In the present article, we report details on the methodology utilized, and the insights, ideas, and prototype solutions we generated using social media data analysis, a survey, and workshops.

## Materials and Methods

The study was led by an interdisciplinary team at our 600-bed academic teaching hospital, Bambino Gesù Children's Hospital (OPBG), in Rome, Italy, funded by EU Horizon 2020 framework under the i-Consent Project (Grant Agreement 741856), in collaboration with LUMSA University i-Consent unit in Rome.

Principles from IDEO's design thinking approach (31) were blended with a mix of qualitative and quantitative research methods to generate inclusive informed consent prototypes for vaccine clinical trials targeting pediatric patients.

**Figure 1:**
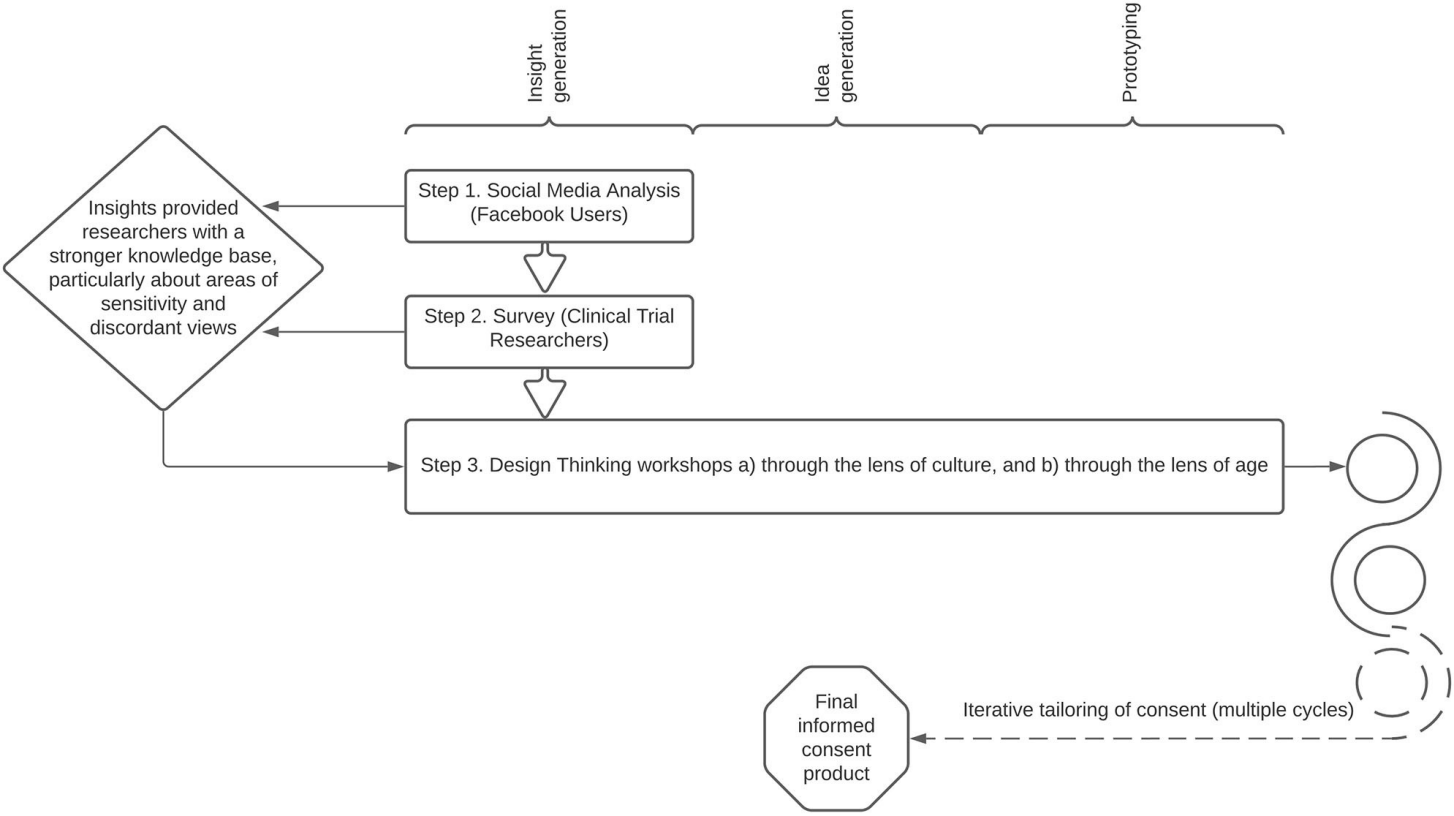
A blended research design approach was used to generate informed consent insights, ideas, and prototypes.

Insight generation was conducted through social media analysis, a survey, and workshops, focused on the lenses of culture and age. Workshops also included idea generation and prioritization, and prototyping phases (Figure 1). The value of the proposed methodology for improving informed consent was discussed with experts in informed consent during meetings of the i-Consent project.

### Ethics Statement

Design thinking activities were conducted in compliance with the ethical requirements set out in i-Consent Project (Grant Agreement 741856) work package 6 (“Ethics requirements”). Workshop and survey participants provided informed consent that was prepared in accordance with the project ethical requirements. Facebook data was acquired from posts with public privacy settings.

### Social Media Analysis

Design Thinking frequently seeks the perspectives of users at different ends of a spectrum (32, 33). We therefore wanted to use natural language processing to rapidly identify posts on public Facebook pages that would give us rapid insights relevant for the design of vaccine clinical trials communications.

The Facebook search function was used in April and May 2019 with words for “vaccination” and “children” in English and Italian. Data scientists identified posts that they perceived to be of highest relevance and engagement. Data were retrieved from posts using a Facepager (34) scraper, and stored in an SQL database. Data considered relevant were text, photos, or videos, the timestamp, number of reactions (e.g., comments or likes), and the hierarchical-level of the comment (e.g., 0 for initial post, 1 for first-level comment, and 2 for comments responding to first-level comments). Python 3.6 was used to pre-process and clean data: comments containing only emoticons, stickers, or mentions of users were deleted. Polyglot is a popular natural language pipeline for Python that can assign a polarity score to a given sentence: comments were given a positive score if the polarity score is greater or equal to 1, negative if the polarity score is lower or equal to −1, and neutral if the polarity score was 0. The score was visualized enabling the identification of high polarity (positive and negative), or “extreme” comments. Qualitative thematic analysis was conducted on the most polarized comments. Journalistic analysis was used to follow posts and their trajectory in real-time.

### Survey

An online survey including numerical, categorical, binary, multiple choice, and open questions, on the use of technology for informed consent was developed using Survey Monkey, and the survey link circulated by e-mail to clinical trial researchers within i-Consent consortium network in Spain and the UK in May and June 2019. Participants were asked about how they currently use technology for the communication of information to participants, their perspectives of how technology could potentially be used, how they felt clinical trial participants could benefit from technology, and how they felt about using the technology themselves. Responses were completely anonymized, and were downloaded into an Excel spreadsheet and descriptive analyses were conducted.

### Workshops

#### Workshop Group 1: The Lens of Culture

Parents (*n* = 2), cultural mediators (*n* = 6) with diverse backgrounds, researchers (*n* = 3), clinicians (*n* = 3), and ethics experts (*n* = 2) were recruited from Bambino Gesù Children's Hospital (OPBG), LUMSA University, and Salute Migranti Forzati Roma (SAMIFO), an institution that supports forced migrants to attain healthcare in Rome's health system.

Two workshops were held. In the first, the facilitator encouraged empathy by describing a scenario where a teenage girl was invited to part in a hypothetical vaccine trial of a currently unlicensed human papillomavirus vaccine, and then re-creating the girls' user journey. The group was provided with (a) a hypothetical invite letter and (b) sections of a hypothetical informed consent document related to vaccine receipt, side-effects, sexual health, and data/privacy. Both documents were based upon informed consent materials that had been used at OPBG for randomized clinical trials. Participants were asked to add statements to an empathy map—a collaborative tool inspired by IDEO and developed at Stanford d.school to help synthesize participants thoughts and feelings (35). The facilitator discussed the comments with the group, particularly noting where they converged and diverged, to develop consensus on the priority problems in the document. IDEO brainstorm rules (36) were applied to generate ideas for solutions. After the first workshop, a researcher entered the participants' comments into an excel spreadsheet and conducted qualitative thematic analysis to identify document modifications considered necessary by the participants. The document was then rewritten accordingly.

In the second workshop, participants were divided into 4 groups with the objective to develop wireframe prototypes. Each group was provided with both the old and the modified document text and craft materials, and asked to create a prototype for an improved informed consent for their choice of technology. Each group presented their prototype explaining its structural features and the reasons behind modifications made to the text.

#### Workshop Group 2: The Lens of Age

Four children aged 11–14 (1 with cystic fibrosis, 3 healthy) and four parents were recruited from Bambino Gesù Children's Hospital (OPBG). A role-play was conducted to introduce participants to, and develop empathy for the following persona and scenario: A healthy child who learns about and consents to an experimental vaccine trial, then, after receiving the injection, returns home and develops a low fever. Children were asked to express their feelings through the creation of balloon faces and empathy maps. A selection of tools including specialized applications and bots, as well as video call, voice call, and SMS, were presented to the children as potential options for the delivery of informed consent information. Children were taken along the user journey and asked to vote on which type of multimedia format they would prefer to use at different stages of the scenario. The group was then split into non-familial child-adult pairs, and each pair was asked to create prototype text scripts between the patient and the clinical researcher for their preferred multimedia type.

## Results

### Social Media Analysis

Based on the search conducted through Facebook, 4 posts of high engagement and relevance were selected. Posts were extracted from two major news sites, La Repubblica in Italy and the BBC in the UK, and from the Facebook page of the Italian Society of Pediatrics (SIP). Two posts were extracted from the Facebook page of La Repubblica—two identical posts published one year apart—on the importance of giving vaccines to children. One post was extracted from the BBC Facebook page that was about the link between HPV vaccine and a drop in cervical disease. The last post was extracted from the page of the Italian Society of Pediatrics (SIP) and it was about the importance of getting vaccinated against pertussis during pregnancy.

**Figure 2:**
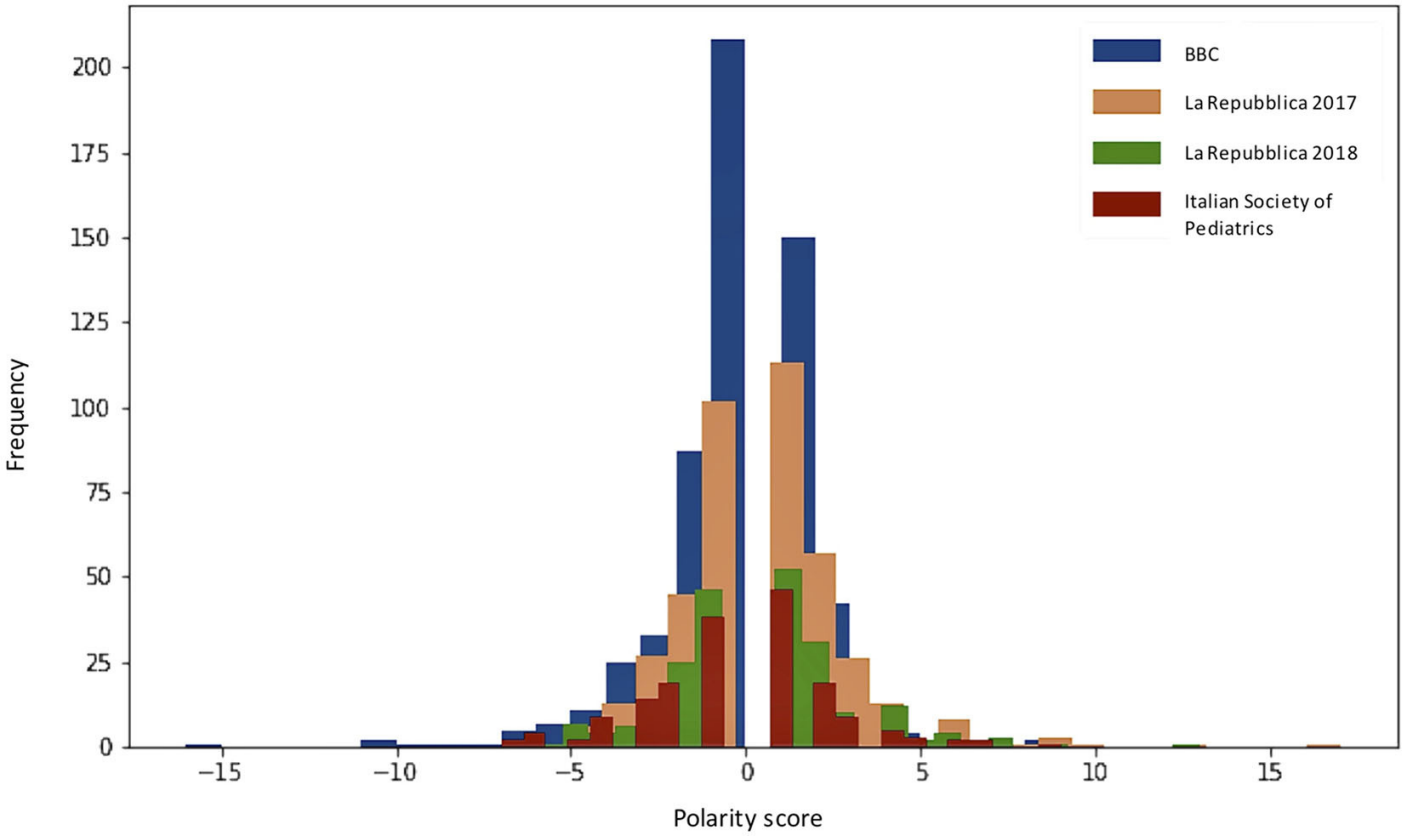
Polarity of comments for each post.

Descriptive characteristics, extracted using NLP, and polarity scores of the selected posts are presented in Table 1. The polarity distribution of the comments as determined by NLP is provided in Figure 2. By following the ISP post journalistically, it was evident that reactions were positive until it was re-posted onto the page of an anti-vaccine group, upon which comments became strongly negative.

**Table 1:** Descriptive characteristics of four selected Facebook posts in Italy and the UK.

**Facebook page**	**La Repubblica**		**BBC**	**Italian Society of Pediatrics**
Topic	The importance of giving vaccines to children		The link between HPV vaccine and a drop in cervical disease (text)	The importance of getting vaccinated against pertussis during pregnancy
Format	Text and video		Text	Text
User interaction mode	Passive		Passive	Active: invited pregnant women to get a vaccine and take and post a selfie.
Date of post	8 May 2017^1^	8 May 2018^2^	4 April 2019^3^	23 April 2019^4^
Reactions (*n*)	2.1 k	2.3 k	22 k	287
Comments (*n*)	988	369	1.7k	404
Shares (*n*)	852	737	6.6k	678
Average polarity score	0.27	0.48	−0.8	−0.12
Proportion of positive comments (%)	53.6	56.6	35.4	49.7

1 *https://www.facebook.com/179618821150/posts/10155487940361151?sfns=xmwa*.

2 *https://www.facebook.com/179618821150/posts/10157752802101151?sfns=xmwa*.

3 *https://www.facebook.com/228735667216/posts/10156595513227217?sfns=xmwa*.

4 *https://www.facebook.com/295443760472888/posts/2643171269033447?sfns=cwmo*.

Positive and negative comments with the most extreme values were then selected and compared qualitatively to enable researchers to quickly understand where perspectives were concordant and discordant between people with extreme views and build their understanding of the types of topics that they may want to focus on during the design workshops.

An example of concordance on the BBC HPV vaccine post was that both positive and negative extremes said that they would let the daughter decide for herself. An example of discordance was “The vaccine was pretty new I didn't know whether it would work or not I didn't know what kind of reaction she would have to it” from the positive extreme, and “Most cervical cancer is not caused by this virus. In fact there has never been a cause and effect relationship between this virus and cancer—only correlational data” from the negative extreme.

### Survey

Thirty Clinical Trial Researchers from Spain (*n* = 23) and the UK (*n* = 7), working in the public (*n* = 22), private (*n* = 1), and both public and private (*n* = 7) sectors, gave their consent to and completed the survey. Seventeen were male and 13 female, and the median age was 55.5 years (range 33 to 69 years). Eighty seven percent (*n* = 26) were medical doctors, 10% (*n* = 3) were nurses, and one respondent did not provide an occupation. The median number of trials respondents had worked on was 6.5 (range 1 to 60). Five respondents were members of ethical boards.

The majority of respondents reported that they do not currently use technology to communicate information to research participants at any point in research studies. 36.7% use technology before the study, 40% during the study, and 16.7% after the study. Only 10% of respondents used technology to attain an electronic signature.

The vast majority (95.5%) of respondents did not think that new technologies would compromise patient safety. 45.5% (*n* = 10) agreed with the statement “I think that new technologies have the potential to speed up the informed consent process”, while 45.5% (*n* = 19) were unsure and 9% (*n* = 2) thought that technology would slow it down. 78.3% (*n* = 18) of respondents reported that they thought that technology would be beneficial for standardization.

**Figure 3:**
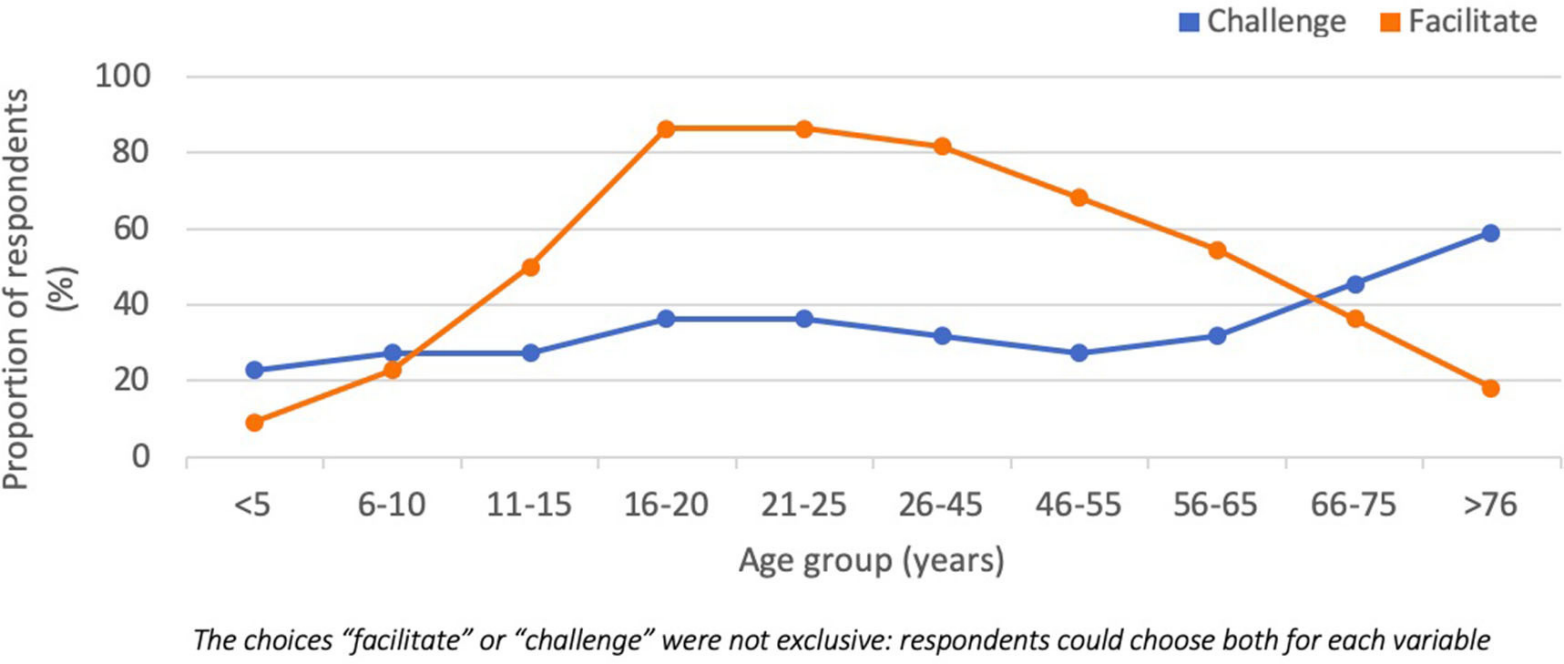
Respondent perspective on whether technology would either facilitate or present a challenge for communication of information with research participate of different ages.

When asked “will technology present a challenge or facilitate the communication of information with different groups of research participants?” respondents perceived that most age-groups would benefit from using technology although challenges were perceived to be bigger than benefits for the <5 years and >75 years age groups (Figure 3). For people with disabilities, respondents perceived that overall, technology would help facilitate communications with people with movement, communication, and social relationship disabilities. More respondents considered that technology could be challenging than facilitating for people with vision, hearing, thinking, learning, mental health, and remembering disabilities. Respondents also considered that technology would present a net-challenge for low income groups, but that it would facilitate communication in medium and high-income groups.

### Workshops

#### Workshop Group 1: The Lens of Culture

Participants highlighted that to enable autonomy, having sufficient access to information and time to digest it were crucial. The complexity of the science and language in the original documents was emphasized, and parents noted that they would seek information from a variety of other sources in addition to the original document. The importance of trust was emphasized—participants wondered if their child had been selected on purpose for the study or if participation in the study had been offered generally. A central issue in the discussion was how poor communication due to differences in cultural background between researchers and participants could risk undermining participants' autonomy. The role of a cultural mediator as a third person in the researcher-participant dynamic was considered, particularly in the context of ensuring a woman's voluntariness in cultures where roles are strongly defined by gender.

Concerns raised were common to all participants, regardless of cultural background. Fears around vaccine side-effects, unknown allergies and sexual health generated the most concern. A list of the main problems and corresponding needs identified by participants are provided in Table 2.

**Table 2:** Problems and needs as identified by participants.

**Identified problem**	**Identified need**
Complex language	Clear language
Unclear scientific content	More background on the specifics e.g., how a virus can cause cancer, how vaccines work, reasons for reactions, reasons for children requiring vaccination at an age when most are not sexually active, and reasons for the mention of contraception (required for participation if sexually active)
Unclear study purpose	Explanation of who the researchers are Better explanation of the aim
Unclear process, particularly: • Recruitment method • Role of the child • Study withdrawal • Data access, storage time and anonymization	Description of who the study is targeted at and how the individual was selected • Clear information about the process for both the parent and child • Detailed information about the withdrawal process • More details on the practicalities: when and how data can be accessed by the participant, how long it will be used, sharing with third parties, and how data could potentially be linked back to participants
Insufficient information about alternatives to trial participation	More explicit details about the other options available outside of the study (for example other HPV vaccines available in Italy).
Insufficient information about risks, particularly: • Vaccine side effects and how long they may last • Allergies, particularly unknown ones • Any potential risks to fertility	More, and clearer information about risks, and about allergies including if and how symptoms for any unknown allergies may have presented previously
Unbalanced information (i.e., too much information about privacy)	Balance the sections of the informed consent form in line with the patients concerns (less privacy, more health risks)
Coercion through guilt (altruism was noted as a reason for study participation in the invitation letter)	Clearer, more balanced information about benefits and risks (including separation of individual and societal)
The need to seek information from other sources was expressed	Guidance to good sources of information
Difficult conversations to have in the family unit	Provide materials for communicating with a child on the topic; or communicating with other family members (e.g., husband)

**Figure 4:**
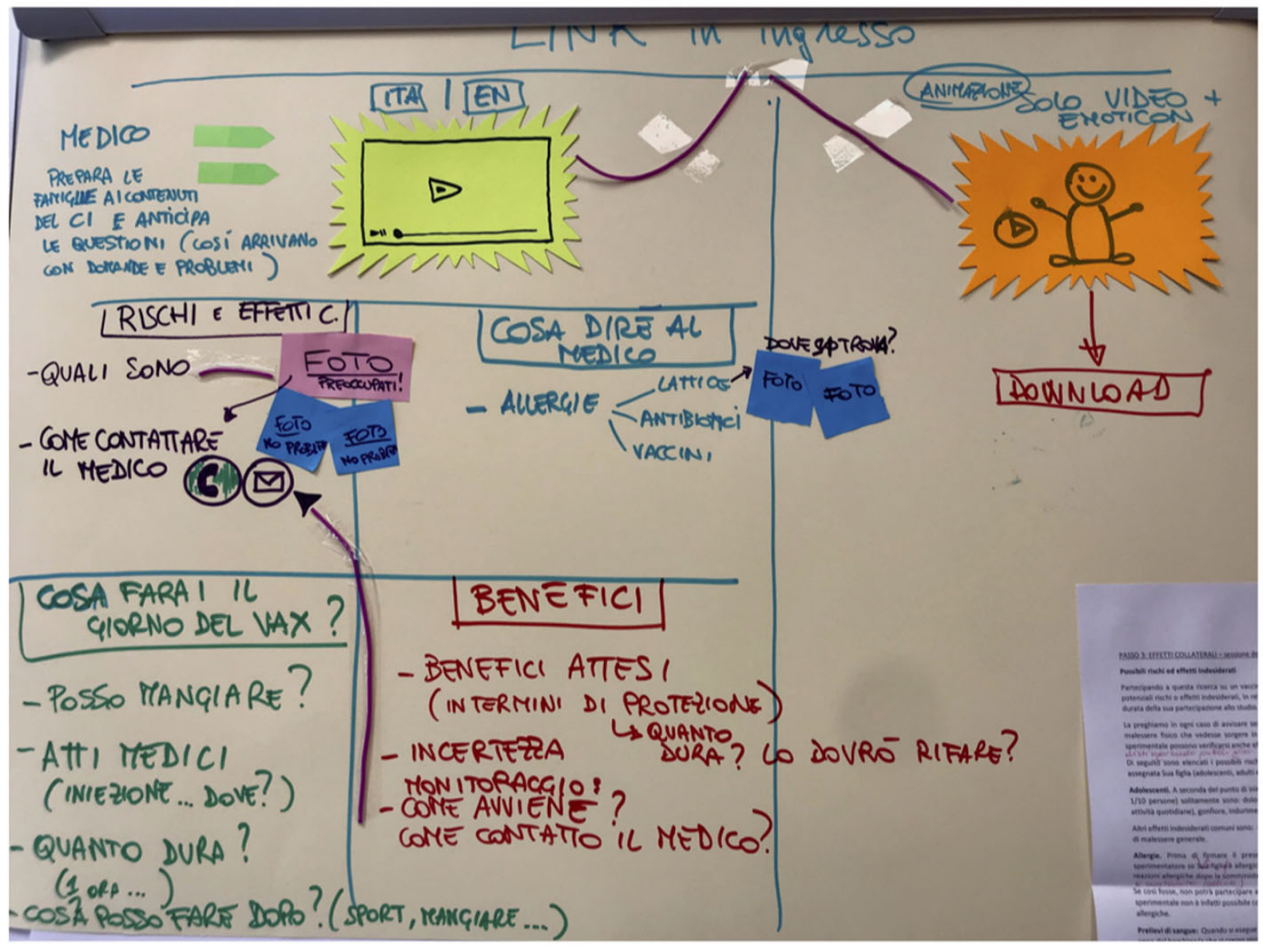
An example of a wireframe prototype on paper.

Each of the four participant groups was allocated a topic, and developed a paper prototype (an example is provided in Figure 4) for an interface of their choice. An overview of the prototypes is provided in Table 3.

**Table 3:** An overview of the prototypes developed.

**Prototype group**	**Interface**	**Overview of structure**	**Overview of content**	**Overview of media**
1. Side effects	Website—main page links to either a child or an adult's site	Child. One page	Basic child's site (one page) containing a video for download	Animation with emoticon
		Adult. Page split into 5 main (linked) sections with link to a sub-page detailing side effects	General information/FAQ	Video (multilingual)—doctor providing information
			Risks and side effects of vaccination	Text description with LINK to photos; contact details of doctor (phone/e-mail)
			What to say to the doctor (allergies)	Text description and photos
			What to do on the day of the vaccination	Text description
			Benefits of vaccination	Text description; LINK to “contact doctor”
2. Privacy	Mobile phone app	5 (linked) pages	Why is your consent important?	Text
			Who is holding my information?	Text and link to OPBG and vaccine society websites
			Are you free to withdraw from participating?	Link to details on how to withdraw
			Can I have access to my data?	Text and link to OPBG
			Check that everything is understood and consent	Checkbox
3. Vaccine administration	Paper or phone app	Text split into 5 sections, and calendar	Introduction explaining where the vaccine is being administered	Text
			When and how is the vaccine administered?	Text
			How will analyses be done?	Text
			Calendar	Calendar
			Next contact with researchers	Text
4. Sexual health	Paper	Text with 5 headings	What is HPV?	Text
			The vaccine	Text
			Who should be vaccinated?	Text
			Contraindications	Text
			To participate in the study	Text

#### Workshop Group 2: The Lens of Age

It became apparent that many opportunities exist to improve the informed consent process, in different areas of the information ecosystem and with different technologies. Participants selected different communication strategies for different severities of disease: they wanted to call a doctor in an emergency, text for less severe health issues, and use an automated form for general Q&A. From observing interactions between the children and parents, we learned about the family dynamic. The parents assumed an authoritative role in the situation, putting the child patient in a position of less power and autonomy, even though our age range went up to 14 years. We learned which words were not understandable to either the children or their parents, what information they found useful and what they were not interested in knowing. The parents were formal in their communication approach with the doctor—the children more direct.

As researchers, we realized it was necessary to think a lot more broadly about the communication ecosystem than had been done previously. The exercise helped generate a range of ideas from comics, videos, apps, games (board and tech), props (vaccine dolls), through to explore rooms, and websites; highlighting the huge range of possible approaches that could be used to improve communication for informed consent in any setting. When we began to consider desirability, feasibility, and viability, it was quickly apparent that these were context specific, and the “best” choice of approach would vary by setting.

## Discussion

We applied a mixed-methods participatory approach that blended research and design methodologies to address the complex problem of tailoring informed consent for a vaccine clinical trial in our setting, a tertiary pediatric hospital in Rome, Italy. The insights we gained complemented the i-Consent consortium's existing knowledge base, and helped us in the process of developing consent materials.

As is stated in many ethical guidelines and other studies, obtaining informed consent should be an ongoing process in clinical research (37, 38) particularly for minors (39). Rather than confining consent to a singular decision or event, we considered informed consent as a continuous, bidirectional communication process that begins at the first contact with the potential participant and continues until the end of the study. We do acknowledge however that for some other scenarios, including neonatal research or emergency consent, the long informed consent process that we envisaged would not necessarily apply (40).

To our knowledge, design thinking has only been used in one other study for the development of the informed consent process. Specifically, DT was used to redesign human research protections, for which informed consent was considered as one of many elements of interest (41). Other studies have explored the adoption of participatory methods for developing informed consent. Recently, a crowdsourcing approach was used to highlight the limits of the informed consent used for HIV clinical trials and propose alternative formats, through workshops that, however, were not based on design thinking (42).

We focused on inclusivity by design (through the lenses of age and culture) to improve participants' comprehension and therefore autonomy, which is crucial to the fulfillment of legal and ethical requirements. Usually, informed consent is not designed with users and frequently does not account for needs which may be based upon factors including education, and societal and cultural differences. We share the widely held view that user participation is of paramount importance to good design, and found that workshops based upon design thinking principles provided us with a good platform for involvement. The participatory approach we took enabled us to modify the content of existing consent materials to the communication needs of users, and generate a variety of prototypes for new tools that patients considered valuable to improve their understanding of all of the information necessary to make an informed decision about clinical trial participation.

Triangulation of methods enabled us to gain a more multidimensional, holistic perspective on the problem, as has been described in other studies (43). Through social media analysis, researchers were able to gain a greater understanding of beliefs, attitudes, and misunderstandings in the community.

Social media analysis has previously been used for investigating the discourse on vaccines on the web (44). Qualitative and quantitative analysis of social media data can provide insights on perceived benefits and on misconception on vaccines, which can serve as a knowledge base to plan the dialogue with patients in the informed consent process for vaccine clinical trials. Natural language processing-based techniques can also be used to identify and analyse social media posts on other drugs or clinical procedures for which an informed consent process is requested. We consider that natural language processing could potentially be a helpful tool to facilitate monitoring changes in community sentiment over time (45), through polarity indicators for example. We attained polarity scores for different posts, but without a larger sample size and validation, these scores remain somewhat arbitrary. We propose that if a baseline were generated with these indicators, this technique could potentially be used to monitor the “temperature” of a topic in the population over time, and that this could be an interesting area for future research.

At present, social media analysis is usually based on text extracted from posts that are openly available to the public, and, given the peculiar nature of this kind of research, no consent is asked to the authors of the posts. Ethical aspects on the use of digital data, especially regarding minors, have recently been addressed in a scoping review (46), which highlighted a degree of uncertainty regarding the ethics of this kind of research, and suggested the use of participatory and co-production practices to address these issues together with the population targeted by each study.

A survey provided insights on the perspective and attitudes of researchers to using technological tools for informed consent. While the use of technology or innovative tools for the consent process was limited among the participants, most respondents deemed the use of technology for informed consent as safe, which suggests a potentially high level of acceptability of innovative methods for consent. On the other hand, almost half of respondents were uncertain about the potential of technological tools to speed up the informed consent process. This result points out that more research is needed on the impact of innovative solutions on the time for acquisition of informed consent, which is a rarely considered dimension.

Researchers surveyed identified some groups as potentially finding technology challenging, for example very young children or patients with vision or hearing disabilities. While it was considered that technology could present a challenge to some of these users, it is immediately evident that technology could be a big enabler for access: for example, for those with visual disabilities a range of options including text-to-speech could be utilized whereas those with hearing disabilities would benefit from more visual content. The result might be more indicative of the survey respondents personal experience—the technology they use on a day-to-day basis—than the full range of what is actually available on the market. In any case, this result suggests, on one hand, the need to include people with disabilities in the development of informed consent processes and, on the other hand, the need for more specific education of researchers on the potential of technology for improving accessibility to information for people with disabilities.

Based on the insights gained through the social media analysis and the survey, particularly about potential areas of sensitivity and discordant views, researchers were better prepared to facilitate balanced dialogue from different perspectives during the workshops. In the workshops, while it was clear that needs differed between adults and children, it was also clear from involving participants from different cultures that core human needs such as autonomy were the same for all informed consent users regardless of perceived cultural differences.

While we were able to make immediate modifications to the content of the existing informed consent documentation in our setting, we didn't develop final products for the prototypes of the more disruptive and innovative ideas generated. While we consider that these ideas would enable us to implement informed consent with more impact, their deployment would necessitate process change and our ability to deploy them is highly dependent upon other decision-making factors including available funding. Improving communications in the recruitment phase does have the potential to have reap returns on investment given that randomized controlled trials are very costly to implement, and many do not meet their recruitment targets (47–49).

We feel that this approach gave us an excellent basis upon which to move forward: with the patient's needs and wants clearly stated, we can now scope the existing products on the market in a more targeted way. For the products that do not yet exist, these prototypes provide us with clarity, and therefore a stronger basis upon which we can begin discussions and form collaborations with partners to develop final products. The approach that we developed in Rome has already been utilized by our i-Consent consortium partner, FISABIO in Valencia, Spain, to develop an informed consent communication tool tailored to context (50).

This work has taken on additional relevance since the emergence of SARS-CoV-2 because research on novel vaccines has accelerated globally, and children will need to be included in vaccine trials (51). Vaccine trial recruitment can be affected by potential participants' existing beliefs and misconceptions, and vaccination is a sensitive and politicized issue for which members of a community can have polarized views, particularly with the advent of the internet and social networks (52). Beliefs and misconceptions about vaccines are not static in the community, and our scientific understanding is also progressing at a rapid pace—particularly in the context of coronavirus. Informed consent will need to evolve over time along with the information ecosystem, and we consider that an approach inspired by design thinking could enable rapid adaptation to local needs to be conducted to keep pace.

## Conclusion

We propose using a participatory, mixed-methods approach to design informed consent so that it is better adapted to patients' needs. Such an approach would be helpful in better addressing the needs of different segments of the populations involved in clinical trials. Further evidence should be gained about the impact of this strategy in improving recruitment, decreasing withdrawals and litigations, and improving patient satisfaction during clinical trials.

## Author Contributions

SJ, MD, SLP, and AET all made substantial contributions to the conception or design of the work, the acquisition, analysis or interpretation of data for the work, and drafting the work or revising it critically for important intellectual content. FG reviewed the original manuscript, improved its final structure, and helped developing the discussion.

## Conflict of Interest

SLP was employed by the company AND Consulting Group. The remaining authors declare that the research was conducted in the absence of any commercial or financial relationships that could be construed as a potential conflict of interest.
